# Elimination of Hepatic Rodent *Plasmodium* Parasites by Amino Acid Supplementation

**DOI:** 10.1016/j.isci.2020.101781

**Published:** 2020-11-06

**Authors:** Patrícia Meireles, Daniela Brás, Diana Fontinha, Ângelo F. Chora, Karine Serre, António M. Mendes, Miguel Prudêncio

**Affiliations:** 1 Instituto de Medicina Molecular João Lobo Antunes, Faculdade de Medicina, Universidade de Lisboa, Av. Prof. Egas Moniz, 1649-028 Lisbon, Portugal

**Keywords:** Physiology, Parasitology, Diet

## Abstract

*Plasmodium* parasites, causative agents of malaria, scavenge host nutrients to sustain their intracellular replication. Modulation of the host's nutritional status can potentially help control infection by limiting the parasite's access to nutrients, or by boosting the immune system. Here, we show that dietary supplementation of mice employing a combination of arginine (R) with two additional amino acids, lysine (K) and valine (V), termed RKV, significantly decreases *Plasmodium* liver infection. RKV supplementation results in the elimination of parasites at a late stage of their development in the liver. Our data employing genetic knockout mouse models and *in vivo* depletion of specific cell populations suggest that RKV supplementation boosts the host's overall innate immune response, and that parasite elimination is dependent on MyD88 signaling in immune cells. The immunostimulatory effect of RKV supplementation opens a potential role for dietary supplementation as an adjuvant for prophylaxis or immunization strategies against *Plasmodium* infection.

## Highlights

• RKV supplementation leads to the elimination of hepatic *P. berghei* parasites
• An overall activation of the innate immune system mediates parasite elimination
• MyD88 is a key player in the elimination of hepatic *P. berghei* parasites

## Graphical Abstract

**Figure:**
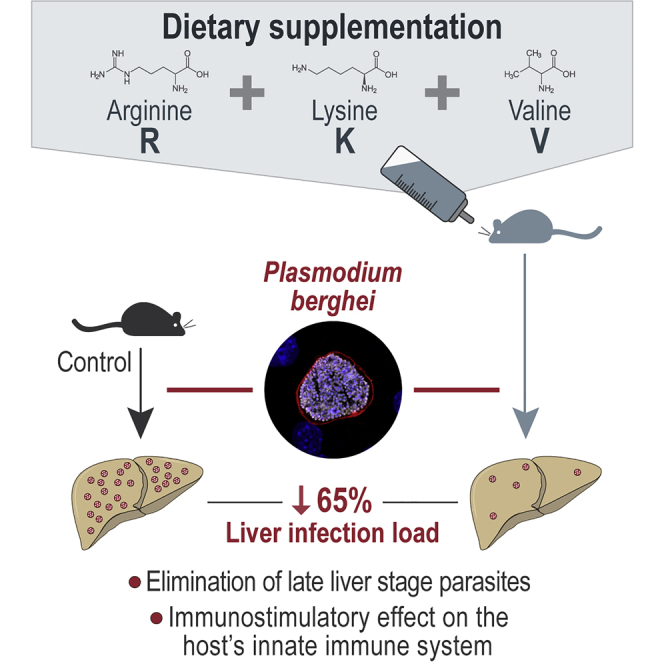

## Introduction

Malaria is an infectious disease that remains a major cause of morbidity and mortality worldwide, for which new cost-effective interventions are urgently needed (WHO, 2019). *Plasmodium* parasites, the causative agents of malaria, are transmitted by female *Anopheles* mosquitoes as sporozoites, which are deposited under the mammalian host's skin and home to the liver through the circulatory system. After traversing several cells, sporozoites productively invade hepatocytes, inside which they develop into exoerythrocytic forms containing thousands of merozoites. The end of the liver stage of *Plasmodium* infection is marked by the release of these newly formed parasites into the bloodstream, where they invade red blood cells, and initiate the symptomatic, erythrocytic stage of the disease (Prudencio et al., 2006).

Numerous studies suggest that poor nutritional status or nutrient deficiencies increase a population's vulnerability to infections (Schaible and Kaufmann, 2007; Jones and Berkley, 2014). That is also the case for malaria, for which it is well established that host deficiencies in several micronutrients (e.g., vitamin A and zinc) can exacerbate malaria, and that modulating parasite access to other nutrients, such as glucose, vitamin B5, and choline, can have a significant impact on parasite growth and, consequently, on disease (Kirk and Saliba, 2007; Mancio-Silva et al., 2017; Counihan et al., 2017; Shankar, 2000; Caulfield et al., 2004). Dietary supplementations employing various nutrients, such as Coenzyme Q10, Vitamin C, Vitamin D, iron, Arg, tetrahydrobiopterin (BH4), or folate, among others, have been shown to directly impact *Plasmodium* erythrocytic stages (Nyariki et al., 2019; Qin et al., 2019; Wu et al., 2018; Castberg et al., 2018; Goheen et al., 2017; Awasthi et al., 2017; Alkaitis and Ackerman, 2016; Meadows et al., 2015). Interestingly, cysteamine has been shown to potentiate the activity of anti-malarial drugs, like artemisinins (Moradin et al., 2016), opening a potential new pathway to using nutrient supplementation to improve malaria treatment. Despite numerous studies to understand how different nutrients may affect *Plasmodium* infection, their usefulness as modulators of disease remains largely unexplored.

Conversely, little is known about the effects of dietary supplementation on the liver stage of *Plasmodium* infection. Dietary supplementation of n-3 fatty acids in the form of fish oil has been shown to inhibit *P. berghei* hepatic development (Vreden et al., 1995). Also, the administration of a high-fat diet to mice highly impaired *Plasmodium* liver infection leading to parasite elimination, an effect associated with increased expression of oxidative stress-related genes (Zuzarte-Luis et al., 2017). Interestingly, iron supplementation has yielded contradictory results in what concerns its impact on *Plasmodium* liver infection. While one study has suggested that it promotes hepatic parasite development (Goma et al., 1996), another, more recent, study reported a hepcidin-dependent decrease in hepatic parasite numbers following iron supplementation (Ferrer et al., 2016). Thus, a more comprehensive understanding on the impact of dietary alterations on the liver stage of *Plasmodium* infection is clearly warranted.

Arg (R) is involved in many metabolic pathways, including the synthesis of nitric oxide (NO), which plays an important role in the killing of invading pathogens, and the synthesis of polyamines via the arginase pathway, which, in turn, can support pathogen growth (Wanasen and Soong, 2008; Das et al., 2010). The competition between these two pathways has been shown to dictate the outcome of infections by *Trypanosoma spp*., *Leishmania spp.*, *Toxoplasma gondii*, *Shistosoma mansoni*, *Candida albicans*, *Helicobacter pylori,* and *Plasmodium spp.* (reviewed in (Das et al., 2010; Phillips, 2018)). Arg is the only amino acid-based dietary supplementation that has been evaluated in the context of malaria. Its administration was reported to increase the circulating levels of Arg in *P. berghei*- and *P. yoelii*-infected mice, leading to enhanced NO production (Martins et al., 2012; Zhu et al., 2012). This, in turn, was shown to significantly impact the pathology associated with the blood stage of *Plasmodium* parasites, reversing cerebrovascular constriction in *P. berghei*-infected mice displaying signs of experimental cerebral malaria (Ong et al., 2018). Yet, while some studies employing *P. yoelii*-infected BALB/c and *P. berghei*-infected C57BL/6 mice report a significant decrease in parasitemia and an improvement in the survival of the animals following Arg supplementation (Zhu et al., 2012; Ong et al., 2018), these effects were not observed in another study employing the latter model (Martins et al., 2012). Therefore, although Arg supplementation has been shown to be beneficial for some aspects of the malaria pathology, the role for this amino acid in the context of the disease remains to be clearly defined.

Our previous studies have shown that Arg uptake plays an essential role in the *Plasmodium* parasite's intra-hepatic development and maturation (Meireles et al., 2017). In the liver, Arg is taken up by the infected hepatocytes through the host cell's SLC7A2-encoded transporters and is metabolized primarily by the parasite's own arginase pathway to secure the biosynthesis of polyamines which are crucial for its development (Meireles et al., 2017). This observation led us to hypothesize that the liver stage of *Plasmodium* infection might be impaired by a dietary supplementation that would significantly alter the parasite's metabolism of Arg. To investigate this, we aimed at blocking the polyamine synthesis pathway of both the parasite and the host cell by providing the amino acids lysine (Lys, K) and valine (Val, V), which are well-known inhibitors of the arginase enzyme (Hunter and Downs, 1945). The blockage of Arg metabolism for polyamine synthesis is expected to channel the use of this amino acid as a substrate of iNOS, boosting NO production and potentially impacting *Plasmodium* development in the liver.

We, therefore, designed an amino acid supplementation regimen named RKV, which combines Arg, R with Lys, K and Val, V and employed the rodent *P. berghei* parasite in combination with different mouse strains to investigate RKV's impact on *Plasmodium* liver infection. Our results show that RKV supplementation leads to a significant elimination of hepatic *Plasmodium* parasites, likely through the action of the innate immune system, and in an MyD88-dependent manner.

## Results

### RKV Dietary Supplementation Impairs *P. berghei* Hepatic Infection

**Figure 1:**
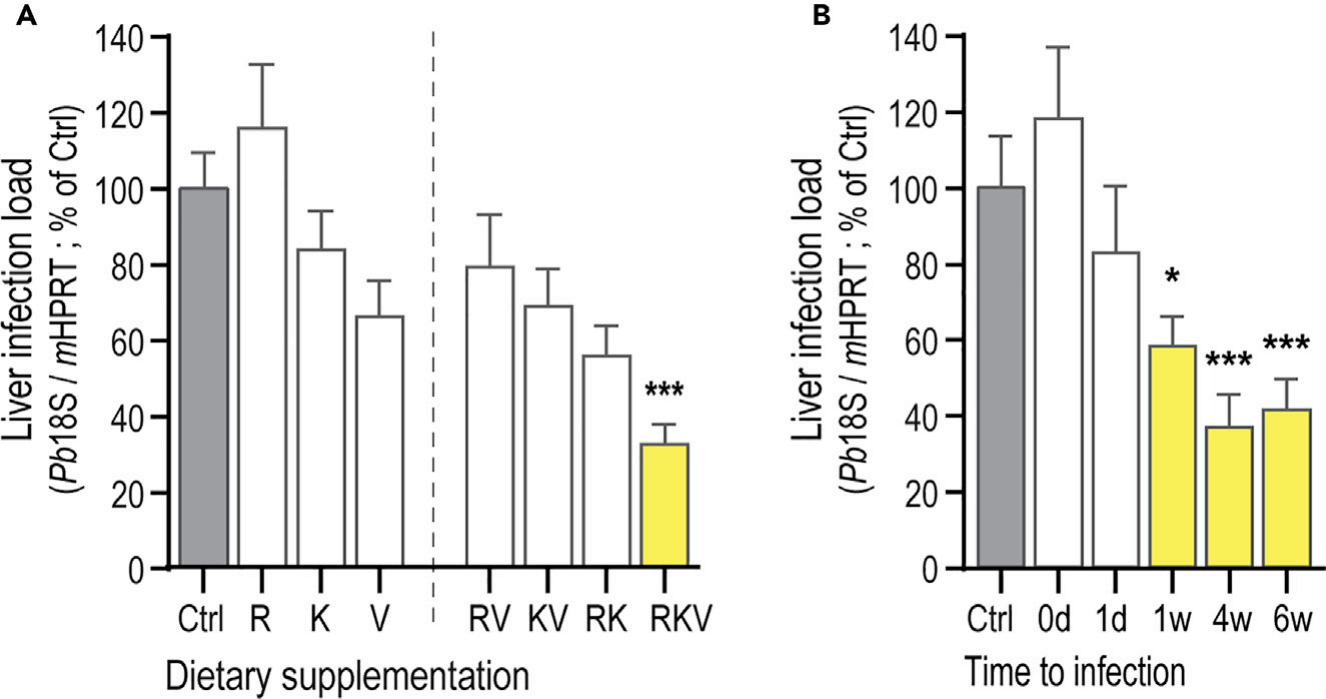
RKV Supplementation Increases Mammalian Host Resistance to *Plasmodium* Liver Infection (A) The drinking water of C57BL/6J WT mice was supplemented with 2.5% (w/v) of single amino acids Arg (R), Lys (K), and Val (V) (left), or with different combinations of two (RK, KV, and RK) or three (RKV) of the same amino acids (right), for 4 weeks prior to infection with *P. berghei* sporozoites. Liver parasite load was assessed at 46 hpi by qRT-PCR. Pool of ≥3 independent experiments. (B) The drinking water of C57BL/6J WT mice was replaced by RKV-supplemented water on the day of *P. berghei* sporozoite injection (0 d), or 1 day, 1 week, 4 weeks, or 6 weeks before infection. Forty-six hpi, livers were collected and liver parasite load was assessed by qRT-PCR. Mice drinking non-supplemented water were used as controls. Pool of 2–5 independent experiments. Statistical significances assessed by Kruskal-Wallis with post-test Dunn applied in (A) and One-way ANOVA with post-test Dunett in (B) with ∗p < 0.05 and ∗∗∗p < 0.001. Significant differences are indicated in yellow. See also Figure S1.

To assess the possibility of modulating hepatic infection by *Plasmodium* through dietary supplementation, we sought to increase the bioavailabilty of Arg (R) as a physiological substrate for the synthesis of nitric oxide (NO), which is a key mediator of immune responses (Lee et al., 2017; Roth, 1992; Bogdan, 2001). To achieve this, we supplemented the drinking water of C57BL/6J mice with 2.5% (w/v) of Arg and the arginase inhibitors Lys (K) and Val (V), either individually or in combinations of equal concentrations (RV, KV, RK, and RKV). Mice were provided with supplemented water *ad libitum* for 4 weeks, while non-supplemented sterilized water was provided to control (Ctrl) mice, following which all animals were infected by intravenous (iv) injection of luciferase-expressing rodent *P. berghei* sporozoites. Our quantitative real-time polymerase chain reaction (qRT-PCR) results show that RKV supplementation significantly decreased *P. berghei* liver load 46 hr post-infection (hpi) by 65 ± 31%, while supplementation with either the individual components or with any combination of two of the amino acids that make up the RKV formulation did not have a significant impact on liver infection (Figure 1A). This observation is in complete agreement with our bioluminescence analysis of infected mouse livers, which indicated a 63 ± 37% decrease in the hepatic load of RKV-supplemented mice relative to untreated controls (Figures S1A and S1B). Of note, we also showed that RKV supplementation impacts liver infection by *P. yoelii*, another rodent malaria parasite, to an extent similar to that observed for *P. berghei* (70 ± 12% decrease; Figure S1C), indicating that this phenotype is not species-specific, at least among rodent malaria parasites. When *P. berghei* infection was allowed to proceed to the blood, no significant differences between RKV-supplemented and control mice were observed in terms of pre-patency time or survival from experimental cerebral malaria (Figures S1D and S1E), as expected from a <90% difference in liver parasite load between the two groups of mice (Siddiqui et al., 2015).

Having established that RKV supplementation can significantly impact *Plasmodium* liver infection, we then sought to determine the minimum period of dietary supplementation required for this effect to be observed, by varying the duration of supplementation prior to infection. Our results show that one week of RKV supplementation is sufficient to observe a significant decrease in hepatic infection by *P. berghei* (42 ± 32%), an effect that is even more pronounced after 4 weeks of dietary supplementation (68 ± 25%), and which appears to plateau thereafter (53 ± 28% reduction in comparison to non-supplemented controls after 6 weeks of supplementation; Figure 1B).

### RKV Dietary Supplementation Leads to the Elimination of Late Liver Stage Parasites

**Figure 2:**
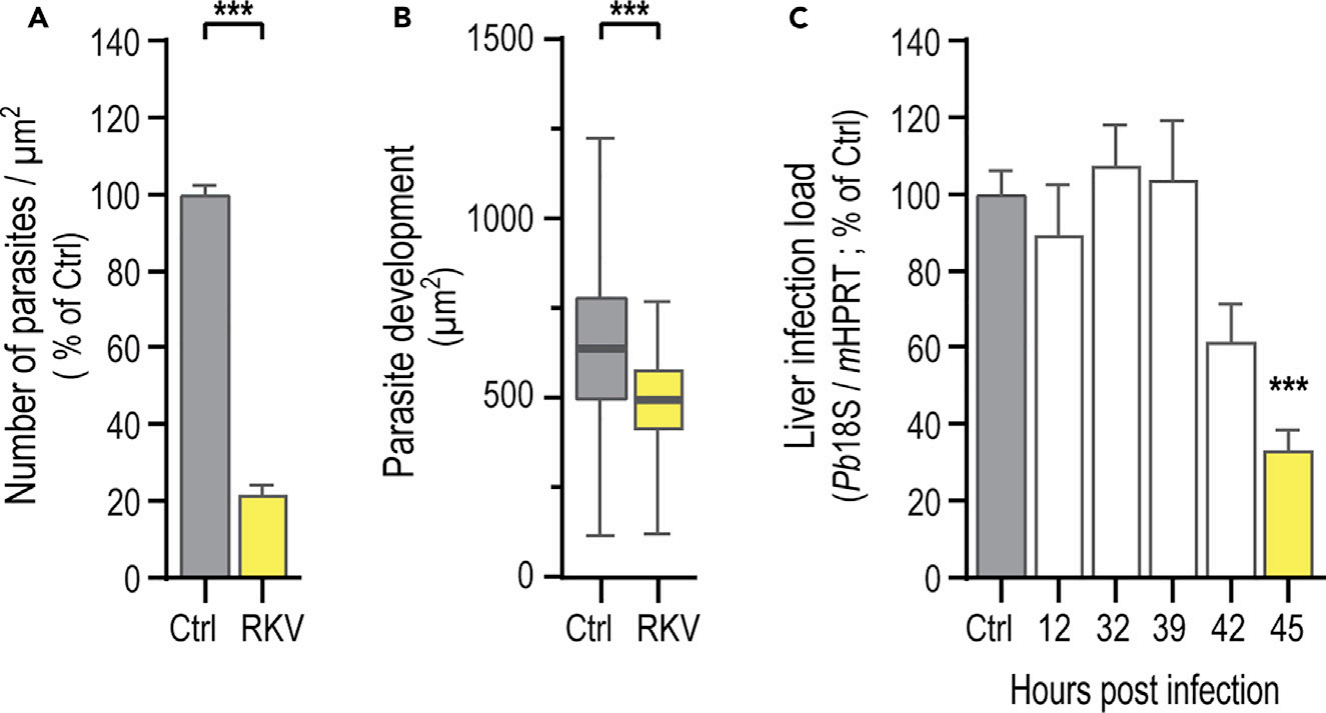
RKV Supplementation Leads to the Active Elimination of Hepatic *P. berghei* Parasites (A and B) The drinking water of C57BL/6J WT mice was supplemented with the RKV combination for 4 weeks prior to infection with *P. berghei* sporozoites and the number (A) and size (B) of liver parasites was assessed by immunofluorescence microscopy at 46 hpi. (C) The livers of Ctrl and RKV-supplemented mice infected with *P. berghei* sporozoites were collected at the indicated timepoints, and parasite liver load was assessed by qRT-PCR. Pool of 2–4 independent experiments with error bars representing SEM. Significant differences established by unpaired t test (A and B) or Kruskal-Wallis with post-test Dunn (C) with ∗∗∗p < 0.001. Significant differences are indicated in yellow.

Having shown that RKV leads to a marked decrease in the liver load of *P. berghei*-infected mice, we asked whether this reduction resulted from a decrease in the number of hepatic parasites and/or an impairment of their intra-hepatic growth. To evaluate both possibilities, liver sections from *P. berghei*-infected Ctrl and RKV-supplemented mice were collected 46 hpi and analyzed by immunofluorescence microscopy. Our results show a marked decrease in the number of parasites per liver area of RKV-supplemented mice relative to controls (Figure 2A), as well as a smaller but statistically significant reduction in parasite size (Figure 2B). The decreased number of parasites suggests that RKV supplementation may lead to either a decrease in hepatocyte invasion by the parasite, or to an elimination of parasites developing in the liver of RKV-supplemented mice. To investigate this, livers from Ctrl and RKV-supplemented mice were collected, and parasite load was assessed by qRT-PCR at different times following injection of *P. berghei* sporozoites. Our results show that the establishment of infection in the liver of RKV-supplemented mice is indistinguishable from that of Ctrl mice, indicating that the parasite's ability to invade and infect hepatocytes is not affected by this dietary supplementation. Notably, parasite load in the livers of RKV-supplemented mice is lower than that of Ctrl mice only from ∼42 hpi onward, suggesting that dietary supplementation leads to the elimination of liver parasites at a late stage of their hepatic development (Figure 2C).

### Impairment of Liver Stage Development Is due to a Direct Effect of Lys (K) Dietary Supplementation on *P. berghei* Parasites

**Figure 3:**
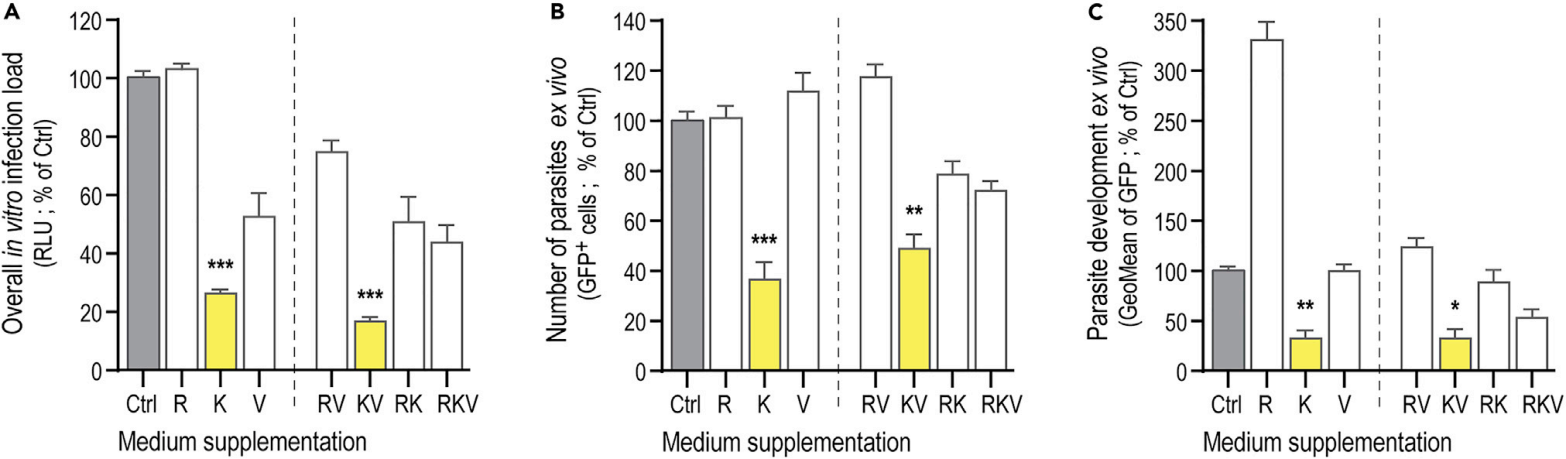
Lys (K) Supplementation Directly Inhibits Hepatic *P. berghei* Parasites *In Vitro* and *Ex Vivo* (A) *In vitro* cultured Huh7 cells were infected with luciferase-expressing *P. berghei* parasites and incubated in medium with the supplementation of either single amino acids, Arg (R), Lys (K), and Val (V), or any combinations of two or three of these ammino acids. Overall infection load was assessed by bioluminescence at 48 hpi. (B and C) Mouse primary hepatocytes were incubated with either single amino acids Arg (R), Lys (K), and Val (V), or any combinations of two or three of these ammino acids, prior to infection with GFP-expressing *P. berghei* parasites. Flow cytometry analysis was used to quantify the relative proportion of infected cells at 48 hpi by assessing the number of GFP^+^ cells (B) as well as parasite development inside hepatocytes by determining the geometric mean of the GFP signal intensity (C). Pools of 2–3 independent experiments with error bars representing SEM. Significant differences established by Kruskal-Wallis with post-test Dunn with ∗p < 0.05, ∗∗p < 0.01 and ∗∗∗p < 0.001. Significant differences are indicated in yellow.

In order to investigate the direct impact of amino acid supplementation on hepatic *Plasmodium* parasites, Huh7 cells, a human hepatoma cell line, were infected with luciferase-expressing *P. berghei* parasites in the presence of high concentration of either the individual components or with the various combinations of amino acids that make up the RKV formulation. Our results show that supplementation of the culture medium with both Lys alone and the KV combination lead to a marked decrease in hepatic infection *in vitro*, which is stronger than that observed with RKV (Figure 3A). To further investigate this, we infected mouse primary hepatocytes with GFP-expressing *P. berghei* parasites, which enable independently assessing the number of infected cells and the extent of parasite development inside these cells by flow cytometry (Prudencio et al., 2008). Our *ex vivo* data clearly show that supplementation with either Lys alone or the KV combination significantly reduces the number of infected hepatocytes at 46 hpi by 63 ± 18% and 50 ± 14%, respectively (Figure 3B), while also decreasing intra-hepatic parasite development by 67 ± 19% and 65 ± 19% (Figure 3C). Also of note, supplementation with Arg alone had no impact on the number of infected hepatocytes (Figure 3B) but markedly increased parasite development at 46 hpi (Figure 3C).

Collectively, these results indicate that, both *in vitro* and *ex vivo*, Lys exerts an inhibitory effect on *Plasmodium* hepatic infection, whereas Arg enhances development, through direct effects on the parasite. Conversely, in mice, co-supplementation of Arg with Lys and Val, but not with Lys alone, leads to a striking decrease in hepatic parasite numbers (Figure 1A), indicating that the elimination of liver parasites *in vivo* occurs in a mammalian organism-dependent manner and cannot be explained solely on the basis of its direct effect on the parasite's metabolism. These observations suggest a potential role for the inflammatory or immune responses on the inhibition of liver infection in the context of RKV supplementation.

### RKV Dietary Supplementation Does Not Induce Liver Damage or Metabolic Inflammation

To assess the impact of RKV supplementation on the health status of the mice and investigate a potential metabolic inflammation of the liver, we started by analyzing several in-life and biochemical parameters in RKV-supplemented and Ctrl mice. Our results revealed no significant differences between the two groups of mice in terms of mouse weight and water consumption, or the array of plasma parameters analyzed (Table 1).

**Table 1:** Mouse Weight, Average Water Intake, and Plasma Biochemistry of Ctrl and RKV-Supplemented Mice

Parameter	Ctrl	RKV	p value
Mouse weight (g)^a^	22.48 ± 1.97	23.45 ± 0.71	0.3527 (ns)
Daily water intake (mL)^b^	4.36 ± 0.33	4.41 ± 0.56	0.9767 (ns)
Serum parameters^c^			
ALT (U per L)	20.33 ± 2.31	14.67 ± 2.52	0.077 (ns)
ALP (U per L)	92.60 ± 20.66	100.73 ± 29.10	0.070 (ns)
AST (U per L)	100.00 ± 5.00	111.33 ± 10.79	0.400 (ns)
GGT (U per L)	1.33 ± 0.58	1.33 ± 0.58	0.792 (ns)
Total protein (g per dL)	3.83 ± 0.21	3.93 ± 0.38	0.700 (ns)
Total bilirrubin (mg per dL)	0.01 ± 0.01	0.04 ± 0.02	0.110 (ns)
BUN (mg per dL)	43.03 ± 10.66	44.95 ± 0.92	1.000 (ns)
Creatinine (mg per dL)	0.22 ± 0.03	0.23 ± 0.05	1.000 (ns)

Data are represented as mean ± SD. p values were determined using the non-parametric two-tailed Mann-Whitney test. See also Figure S2.

ns, not significant; ALT, alanine aminotransferase; ALP, alkaline phosphatase; AST, aspartate aminotransferase; GGT, gamma-glutamyltransferase; BUN, blood urea nitrogen.

a Mouse weight on the day of infection. N = 2 independent experiments.

b Average daily water intake per mouse on the fourth week of supplementation. N > 3 independent experiments.

c Concentration in the plasma at the time of liver collection (45-46 hpi). N = 3 independent experiments.

Furthermore, histological analyses of liver sections from Ctrl and RKV-supplemented mice revealed no alterations in the liver architecture of the latter (Figure S2A), and similar scores of hepatocellular damage and liver inflammatory cell infiltration for both (Figures S2B and S2C). Finally, we analyzed the potential induction of an oxidative stress response in the livers of Ctrl and RKV-supplemented mice by quantifying 16 oxidative stress-related genes, including heme oxygenase-1 (HO-1, encoded by *Hmox1*), an enzyme that has been shown to be upregulated during hepatic *Plasmodium* infection (Epiphanio et al., 2008). Our qRT-PCR data show that the expression of neither of those genes is altered in RKV-supplemented mice in comparison to Ctrl mice (Figure S2D). Overall, our data suggest that a 4-week regimen of RKV dietary supplementation does not bear significant toxicity to the mice or negatively impact their health status.

### The Effect of RKV Dietary Supplementation on Hepatic Infection Is Mediated by the Host Immune System but Not Dependent on NO Production

**Figure 4:**
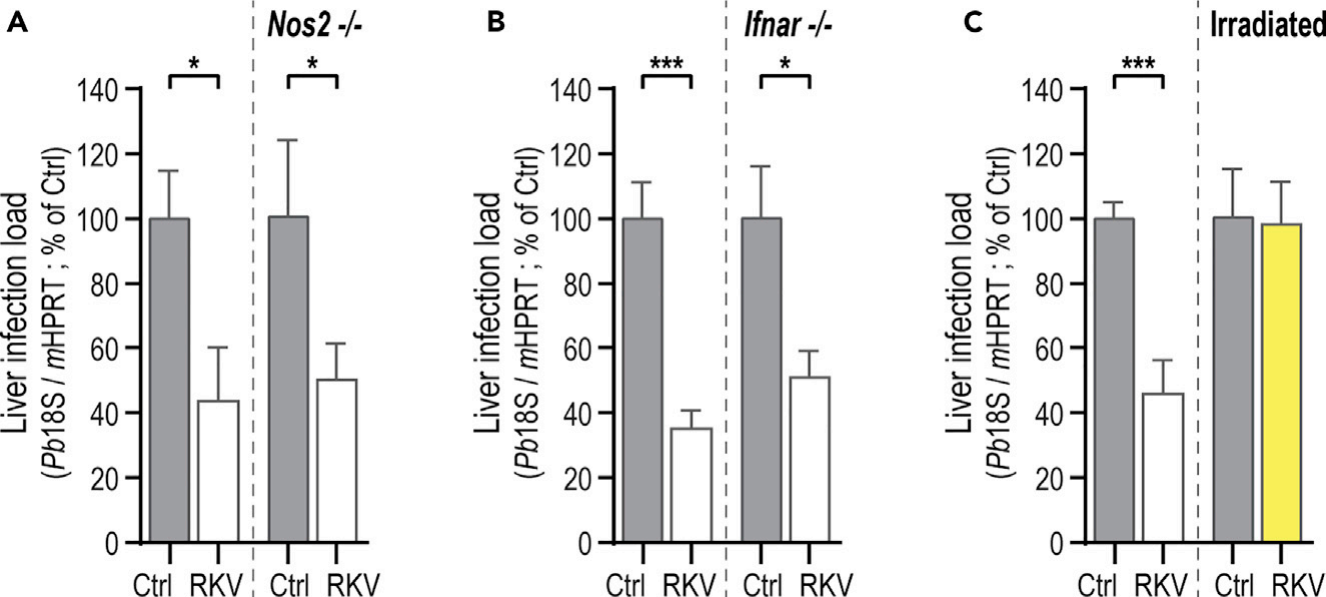
RKV-Dependent Parasite Elimination Is Immune-Mediated but Does Not Rely on NO Production Ctrl and RKV-supplemented mice were allowed to drink *ad libitum* for 4 weeks, after which they were infected with *P. berghei* sporozoites and parasite liver load was assessed by qRT-PCR 46 hpi. (A) Nos2^−/−^ mice were employed to assess whether NO production via iNOS is involved in RKV-dependent hepatic parasite elimination. (B) Ifnar^−/-^ mice were employed to determine whether the RKV-dependent parasite elimination mechanism is dependent on a boost of the type-I IFN response. Pool of >3 independent experiments. (C) WT mice were irradiated with 900 rad one day prior to infection with *P. berghei* sporozoites. Pool of 2 independent experiments. Error bars representing SEM. Significant differences established by two-tailed Mann-Whitney test (A) or Unpaired t test (B and C) with ∗p < 0.05 and ∗∗∗p < 0.001. Phenotype reversion shown in yellow. See also Figure S3.

The rationale for formulating the RKV dietary supplementation arose from the hypothesis that the addition of Lys and Val to an Arg-based dietary supplementation might inhibit arginase activity, consequently channeling the available Arg toward NO production by immune cells, and ultimately leading to parasite elimination. To directly test this hypothesis, we compared the liver infection loads of Ctrl and RKV-supplemented Nos2^−/−^ mice, which cannot produce NO via iNOS. Our results show that RKV dietary supplementation leads to a reduction in liver parasite load for Nos2^−/−^ mice similar to that observed in WT mice (Figure 4A). We further observed that the expression of iNOS in *P. berghei*-infected, RKV-supplemented, WT mice is similar to that of their non-supplemented counterparts (Figure S3A). Overall, these data indicate that parasite elimination upon RKV dietary supplementation does not depend on an increase in iNOS-mediated NO production. Next, we evaluated whether the observed hepatic parasite elimination could be mediated by an immune response elicited or boosted by the RKV dietary supplementation. First, we assessed the impact of RKV-supplementation on Ifnar^−/-^ mice, which lack the type-I interferon receptor, to evaluate the role of type I-IFN innate immune responses in the observed decrease in hepatic parasite survival. This innate response has been shown to peak at around 42 hpi following injection of *Plasmodium* sporozoites and to control liver infection (Liehl et al., 2014, 2015; Miller et al., 2014). Our data showed that the absence of type-I IFN signaling does not abolish the decrease in liver parasite load consistently observed in RKV-supplemented mice (Figure 4B). Moreover, similar expression levels for several Interferon-stimulated genes (ISGs), namely Ifit1, Ifi44, Usp18, Ifit3 and Irf7, were observed in the livers of Ctrl and RKV-supplemented mice at various time points after infection (Figure S3B). Hence, even though a type-I IFN-mediated immune response is active in RKV-supplemented mice, it does not appear to be responsible for the parasite elimination observed. Second, mice that were previously subjected to lethal irradiation, and therefore completely ablated of their immune system (Greenberger and Epperly, 2009; Paix et al., 2018), were supplemented with RKV, in parallel with non-irradiated and non-supplemented Ctrl mice. Our results show that irradiation of RKV-supplemented mice before infection completely abolishes the reduction in liver parasite load observed in supplemented, non-irradiated mice, clearly suggesting an implication of the immune system in parasite elimination upon RKV supplementation (Figure 4C).

### MyD88 Signaling Is Essential for RKV Dietary Supplementation-Mediated Elimination of Hepatic Parasites

To dissect the components of the immune system directly involved in the mechanism of parasite elimination triggered by RKV dietary supplementation, we used a combination of genetic KO mice and specific antibodies to assess the impact of different immune cell populations in the observed phenotype.

**Figure 5:**
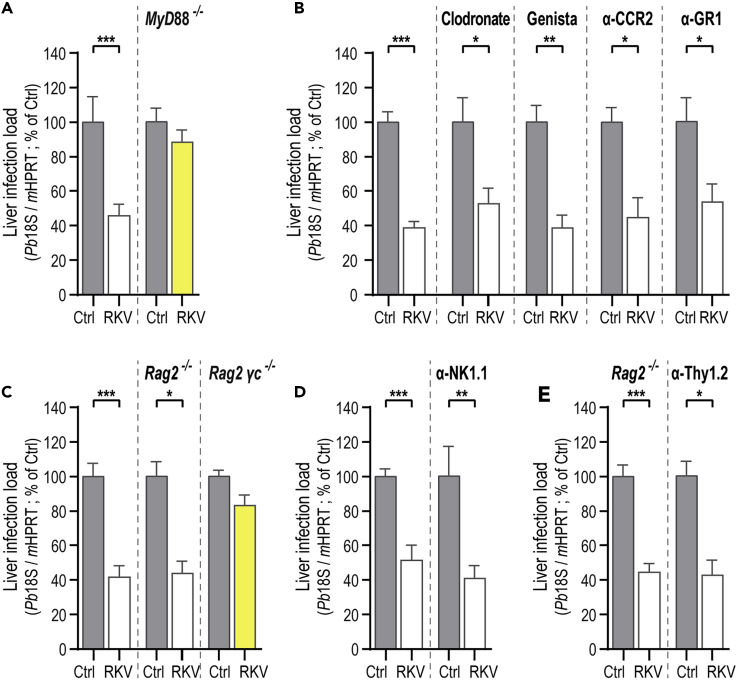
MyD88 Signaling Is Essential for the Multidimensional Stimulation of the Host's Innate Immune System Induced by RKV Supplementation All Ctrl and RKV-supplemented mice were allowed to drink *ad libitum* for 4 weeks, after which they were infected with *P. berghei* sporozoites and parasite liver load was assessed by qRT-PCR 46 hpi. (A) MyD88^−/−^ mice were employed to assess the role of innate immunity and myeloid cells in hepatic parasite elimination. (B) Phagocytes were depleted through the administration of liposome-encapsulated clodronate 2 days before sporozoite injection. The role of neutrophils on RKV-dependent parasite elimination was investigated employing Genista mice, which lack mature neutrophils. Monocytes were depleted by the daily injection of anti-CCR2 antibody from day −2 to day 1 post-infection. Finally, anti-Gr1 was administered to WT mice 2 hr after sporozoite injection, in order to deplete both neutrophils and monocytes simultaneously. Anti-CCR2: 1 experiment; All others: pools of 3 independent experiments. (C) WT, Rag2^−/−^ and Rag2^−/−^ ɣ ^−/−^ mice were supplemented with RKV for 4 weeks before infection with *P. berghei* sporozoites. Rag2^−/−^ mice lack all the adaptive lymphoid populations while Rag2^−/−^ ɣ ^−/−^ mice also lack NK cells and ILCs, which are innate immune populations. Pools of >3 independent experiments. (D) NK cells were depleted through the administration of anti-NK1.1 antibody, 1 day before infection with *P. berghei* sporozoites. Pool of 3 independent experiments. (E) Ctrl and RKV-supplemented Rag2^−/−^ mice were injected with anti-Thy1.2 antibody, 1 day before infection with *P. berghei* sporozoites, to deplete ILCs. One experiment. All panels: Error bars represent SEM. Significant differences established by unpaired t test (A, D, and E) or Two-tailed Mann-Whitney test (B and C) with ∗p < 0.05, ∗∗p < 0.01 and ∗∗∗p < 0.001. Phenotype reversion shown in yellow. See also Figure S4.

As most of the cells that compose the innate immune compartment are of the myeloid lineage, we started by employing MyD88^−/−^ mice, a mouse strain that lacks a crucial adaptor molecule involved in signal transduction after recognition of pathogens by innate receptors, such as Toll-like receptors (TLRs), which are essential for the function of myeloid cells (Warner and Nunez, 2013; Arnold-Schrauf et al., 2015; Akira and Takeda, 2004). Our results show that, in the absence of MyD88, the reduction in liver parasite load that is typically observed in RKV-supplemented mice is completely abolished, suggesting that MyD88 signaling is essential for parasite elimination (Figure 5A). To ascertain whether the MyD88 signaling process responsible for this effect occurred in hepatocytes or in myeloid cells, we performed a similar experiment employing Alb-Cre.MyD88^f/f^ and LysM-Cre.MyD88^f/f^ mice, two mouse strains that lack MyD88 specifically in hepatocytes and in myeloid cells, respectively. Our results showed an impairment of *P. berghei* hepatic infection in either Alb-Cre.MyD88^f/f^ or LysM-Cre.MyD88^f/f^ mice similar to that observed in their WT counterparts (MyD88^f/f^ mice; Figure S4A). Although these results strongly indicate that hepatocytes do not play a crucial role in the observed phenotype, a similar conclusion cannot be taken with certitude in the case of the myeloid compartment, as there is evidence that this KO strategy may not be fully efficacious for the various myeloid cell populations (Clausen et al., 1999; Abram et al., 2014). Therefore, we decided to confirm these results by employing alternative methods to deplete the different myeloid cell populations, including phagocytic cells, such as Kupffer cells and macrophages, as well as neutrophils and monocytes.

We started by administering liposome-encapsulated clodronate to RKV-supplemented mice in order to completely eliminate Kupffer cells and strongly reduce the presence of monocytes/macrophages in the liver, as shown by the reduction of expression of the Clec4f, CD68 and F4/80 markers in the liver (Figure S4B). Importantly, our results showed that depleting phagocytes does not abolish the reduction in liver parasite load typically observed upon RKV supplementation, suggesting that these cells are not involved in the process of RKV-mediated hepatic parasite elimination (Figure 5B). Next, we assessed the involvement of both neutrophils and monocytes, independently or in combination, on the mechanism of parasite elimination by RKV supplementation. Our results show that neither of these innate immune cells play a critical role in the observed reduction of parasite survival. RKV supplementation of Genista mice, a mouse model that lacks mature neutrophils (Ordonez-Rueda et al., 2012), as well as of mice injected with the monocyte-depleting anti-CCR2 antibody, displayed a reduction in liver parasite load similar to that observed in RKV-supplemented WT mice, thus excluding neutrophils and monocytes from playing an essential role in the mechanism of RKV-mediated parasite elimination (Figures 5B and S4C). Furthermore, administration of the anti-Gr-1 antibody confirmed and expanded these results showing that even the simultaneous depletion of both neutrophils and monocytes does not abolish the reduction in liver parasite load observed upon RKV supplementation (Figures 5B and S4D). Collectively, these results suggest a crucial role for MyD88 signaling in the process of RKV-dependent parasite elimination and exclude hepatocytes and the main myeloid cell populations as key players in this mechanism.

### RKV-Dependent Hepatic Parasite Elimination Results from the Coordinated Action of Various Host Innate Immune Cell Populations

Having excluded the involvement of the most abundant myeloid cell populations from the mechanism of parasite elimination by RKV supplementation, we proceeded to investigate the possible involvement of lymphoid cells in this process. To this end, we employed Rag2^−/−^ mice, which lack B, T, natural killer (NK) T cells and γδ T cells (Shinkai et al., 1992; Cording et al., 2018), and Rag2^−/−^γ^−/−^ mice, which, in addition to these cells, also lack NK cells and innate lymphoid cells (ILCs) (Mazurier et al., 1999; Cording et al., 2018). Our results showed that while RKV-supplemented Rag2^−/−^ mice display a reduction in hepatic parasite load similar to that observed in RKV-supplemented WT mice, RKV-supplemented Rag2^−/−^γ^−/−^ mice display a liver parasite load similar to that of their non-supplemented Ctrl counterparts (Figure 5C). These results suggest a possible role of NK cells and/or ILCs in the mechanism of RKV-mediated impairment of liver infection, excluding the main adaptive lymphocyte populations from an involvement in parasite elimination by RKV supplementation. Thus, we next assessed the specific contribution of NK cells to this phenotype, through the administration of the depleting anti-NK1.1 antibody to RKV-supplemented and Ctrl mice. Our results showed that depleting approximately 90% of the NK cells in the liver (defined as NK1.1^+^TCRβ^−^ cells; Figure S4E) does not abolish the reduction in the liver parasite load typically observed upon RKV supplementation, excluding NK cells as the sole player in the RKV-related impairment of hepatic infection (Figure 5D).

Finally, we tested whether the reduction in liver parasite load observed upon RKV dietary supplementation could result from the action of ILCs. To test this hypothesis, we injected the anti-Thy1.2 antibody into Rag2^−/−^ mice, leading to the depletion of around 90% of the ILCs in the liver (defined as CD45^+^Lineage^−^CD127^+^ cells; Figure S4F). However, the liver parasite load in supplemented, ILC-depleted mice did not revert to levels similar to those observed in the corresponding Ctrl mice (Figure 5E), suggesting that ILCs are not, by themselves, responsible for the mechanism of RKV-dependent hepatic parasite elimination.

Collectively, these results suggest that *P. berghei* elimination from the livers of RKV-supplemented mice is mediated by the simultaneous action of several components of the innate immune system, rather than depending on a single population of immune cells.

## Discussion

Nutritional supplementation has long been suggested as a possible strategy to impact the outcome of several pathogenic infections (Read et al., 2019; Jimenez-Sousa et al., 2018; Rautiainen et al., 2016; Steinbrenner et al., 2015; Stephensen, 2001; Gois et al., 2017; Somerville et al., 2016). Recently, dietary alterations have been shown to significantly alter the capacity of *Plasmodium,* the malaria parasite, to replicate in the blood of its mammalian host, altering the clinical outcome of infection (Shankar, 2000, Clinton Health Access Initiative, 2016, Caulfield et al., 2004, (MSF), 2013, Nyariki et al., 2019; Qin et al., 2019; Wu et al., 2018; Castberg et al., 2018; Goheen et al., 2017; Awasthi et al., 2017; Alkaitis and Ackerman, 2016; Meadows et al., 2015; Kirk and Saliba, 2007; Mancio-Silva et al., 2017; Counihan et al., 2017). Less is known about the impact of dietary alterations on the capacity of this parasite to complete the initial stage of its mammalian infection in the liver, and on how targeted modifications of nutritional availability can be employed as infection control tools (Vreden et al., 1995; Zuzarte-Luis et al., 2017; Goma et al., 1996; Ferrer et al., 2016). This study aimed at establishing a dietary supplementation that could be used to modulate the establishment of a hepatic infection by *Plasmodium* parasites. We selected the amino acid Arg and its metabolism as the main targets of our approach, due to its well established impact on the host's immune response to infection by various microorganisms, including *Plasmodium* (Wijnands et al., 2015; Li et al., 2007; Badurdeen et al., 2015; Wanasen and Soong, 2008; Peluffo et al., 2004; Ralph et al., 2008; Appleton, 2002; Awasthi et al., 2017). We show that a novel dietary supplementation, named RKV, based on the combination of Arg with two other amino acids, Lys and Val, known for their capacity to inhibit arginase, leads to the elimination of rodent *Plasmodium* parasites *in vivo* at a late stage of their hepatic development, resulting in a strong overall reduction in liver parasite load.

Our *in vitro* and *ex vivo* data indicate that hepatic infection is inhibited by Lys and enhanced by Arg, through direct effects exerted by these amino acids on the parasite. This is in agreement with the notion that Lys competes with Arg for cellular uptake (Lerzynski et al., 2006) and that arginase inhibition by Lys decreases the availability of polyamines required for parasite development (Meireles et al., 2017), with a smaller but significant impact on parasite numbers. On the other hand, supplementation with Arg does not alter the number of parasites successfully infecting the liver but enhances their development (Figure 3C), likely through an increase in polyamine production, in agreement with (Meireles et al., 2017). However, in an *in vivo* setting, in the presence of a functioning immune system and upon co-supplementation with Lys and Val, but not of Lys alone, Arg contributes to an immune response that leads to the significant elimination of liver stage parasites (Figure 1A). Thus, the small but significant decrease in parasite development observed in an *in vivo* setting (Figure 2B) can be explained by a combined Lys- and Val-dependent direct effect on the parasite, whereas the marked reduction in parasite numbers is explained by the immunomodulatory effect of Arg in the context of additional Lys and Val supplementation. Interestingly, while the addition of Val *in vitro* and *ex vivo* seems to be dispensable, our results show that addition of this amino acid *in vivo* is essential to maximize the impact of supplementation on liver parasite load, as the effect of RK supplementation is not as strong as that observed with RKV.

To the best of our knowledge, this is the first report showing that a specific amino acid combination is able to stimulate the elimination of *Plasmodium* hepatic parasites. Our results suggest that this elimination is dependent on a coordinated response by the innate branch of the immune system. The reduction in liver parasite load typically observed in RKV-supplemented mice is completely abolished, not only in mice subjected to lethal irradiation but also specifically in mice lacking the adaptor molecule MyD88, which are unable to activate the inflammatory signaling pathways downstream of TLRs and IL-1 receptor families (Warner and Nunez, 2013; Akira and Takeda, 2004). Furthermore, phenotypic reversion can also be observed in Rag2^−/−^γ^−/−^ mice, which lack NKs and ILCs in addition to all the B-cell receptor- and T cell receptor (TCR)-containing cell populations, but not on Rag2^−/−^ mice, which only lack the latter cellular subsets. These results exclude the involvement of B and T cells, the main components of the adaptive immune system, as well as NK T cells and γδ T cells, which have innate-like features but are also depend on a functional TCR, from the mechanism of RKV-dependent parasite elimination. Even though our work with KO mouse models points to a critical role for the innate compartment of the immune system in the RKV-dependent impairment of *Plasmodium* hepatic infection, parasite elimination cannot be attributed to either a single myeloid cell population or a single ILC population. Our results using LysM-Cre.MyD88^f/f^ mice and Genista mice, as well as various depleting antibodies, show that the absence of different phagocyte populations, neutrophils and monocytes, did not revert the phenotype associated with the RKV supplementation, while an identical result was obtained for specific ILC populations, namely NK cells and ILCs.

Collectively, our data suggest that RKV supplementation might induce an overall boost of the immune system, ultimately leading to parasite elimination in the liver. Importantly, while supplementation of *P. yoelii*-infected BALB/c mice with Arg alone is sufficient to decrease blood stage parasitemia (Zhu et al., 2012), our results indicate that single supplementation of *P. berghei*-infected C57BL/6 mice with either of the amino acids, Arg, Lys or Val does not lead to hepatic parasite elimination, and only their combination in the RKV formulation significantly decreases parasite load. Moreover, in C57BL/6 mice, the activity of iNOS is not required for RKV-mediated suppression of hepatic infection, excluding a direct role of NO in parasite killing as a result of this dietary supplementation. In fact, although the role of Arg as a modulator of the immune system is well established (Kang et al., 2014; Nieves and Langkamp-Henken, 2002; Bronte and Zanovello, 2005; Peranzoni et al., 2007), promoting antigen presentation in dendritic cells, influencing B-cell secretion of immunoglobulins and modulating T cell metabolism, survival, proliferation, and anti-tumor activity (Li et al., 2019; Liu et al., 2019; Geiger et al., 2016; Werner et al., 2016), the specific complex mechanisms behind its activity are not yet fully understood. In the context of *Plasmodium* infection, treatment with Arg was shown to promote an enhanced Th1 cell response during the early stages of *P. yoelii* blood stage infection in BALB/c mice, and to facilitate the latter's humoral immune response, leading to a significant decrease in parasitemia (Wang et al., 2018). However, the role of NO in this inhibition of blood stage parasite development remains poorly understood, with several studies pointing to an immunoregulatory rather than a direct anti-parasitic role of NO in *Plasmodium* infection, in agreement with our observations (Favre et al., 1999; Legorreta-Herrera et al., 2011; Garnica et al., 2003; Jacobs et al., 1995).

Even though the influence of nutrition on malaria is scarcely understood, targeted nutritional supplementation (e.g. iron and folic acid) is known to modulate malaria immunity and pathology (Shankar, 2000, Clinton Health Access Initiative, 2016, Caulfield et al., 2004, (MSF), 2013). Our work raises the possibility of using Arg-based dietary supplementation as a strategy to stimulate the immune system against hepatic forms of *Plasmodium.* The impact of RKV supplementation on the parasite's hepatic infection supports its evaluation as a potential low-cost, safe, and effective formulation to be employed as prophylactic or adjunctive therapy for malaria (Shankar, 2000; Clinton Health Access Initiative, 2016).

### Limitations of the Study

This study addresses the effect of amino acid supplementation on rodent *Plasmodium* parasites. These results should not be directly extrapolated to human malaria parasites.

To infect animals, infected mosquitos were dissected and injected intravenously into mice, which does not correspond to the natural route of infection.

Mice were irradiated in order to study the role of the immune system on the RKV-mediated inhibition of hepatic infection. This procedure eliminates hematopoietic stem cells and their progeny (immune cells) but also damages other cells in the body, such as erythrocytes and enterocytes, which might affect normal physiological function of the host.

Depleting-antibody treatments were employed to study the role of specific populations of immune cells. These antibodies might have off-targets effects and may lead to the elimination of other cells (which might have residual expression of the intended target receptor).

Finally, only one concentration of each amino acid was used in our supplementation experiments. Further experiments are needed to ascertain if these concentrations are the most effective in terms of anti-*Plasmodium* activity, whilst ensuring the absence of toxicity to the host.

### Resource Availability

#### Lead Contact

Further information and requests for resources and reagents should be directed to and will be fulfilled by the Lead Contacts, Miguel Prudêncio (mprudencio@medicina.ulisboa.pt) and António M. Mendes (antoniomendes@medicina.ulisboa.pt).

#### Materials Availability

This study did not generate new unique reagents.

#### Data and Code Availability

All data generated or analyzed during this study will be available from the lead contacts upon request.

## Methods

All methods can be found in the accompanying Transparent Methods supplemental file.

## Supplemental Information

Document S1. Transparent Methods, Figures S1–S4, and Table S1
