# Supplementary material for: Elimination of Hepatic Rodent Plasmodium Parasites by Amino Acid Supplementation
Source: iScience. 2020 Nov 6;23(12):101781. doi: 10.1016/j.isci.2020.101781 (PMC7689548; doi:10.1016/j.isci.2020.101781)
Supplement: Document S1. Transparent Methods, Figures S1–S4, and Table S1 [file mmc1.pdf]

iScience, Volume *23*

Supplemental Information

Elimination of Hepatic Rodent *Plasmodium* Parasites by Amino Acid Supplementation

Patrícia Meireles, Daniela Brás, Diana Fontinha, Ângelo F. Chora, Karine Serre, António M. Mendes, and Miguel Prudêncio

SUPPLEMENTAL FIGURES AND TABLES

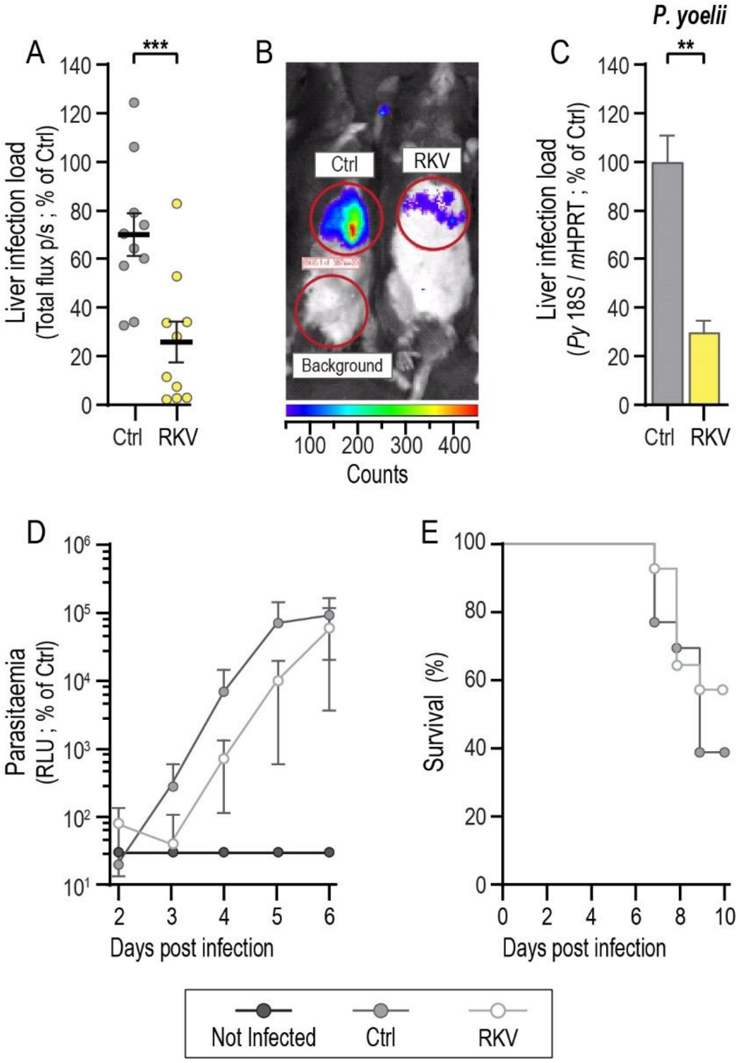

Figure S1 (Related to Figure 1)– RKV supplementation significantly impacts liver parasite load but does not affect blood stage infection or mouse survival. The drinking water of C57BL6 WT mice was supplemented with the RKV combination for 4 weeks prior to infection with luciferase-expressing *P. berghei* **(A, B, D** and **E)** or with *P. yoelii* **(C)** sporozoites. **(A)** Liver parasite load was assessed at 46 hpi by bioluminescence. Pool of 3 independent experiments. **(B)** Representative image of bioluminescence assessment of liver infection in Ctrl and RKV-supplemented mice. **(C)** Liver parasite load in *P. yoelii* -infected mice was assessed at 46 hpi by qRT-PCR. One experiment. **(D)** Blood parasite load in Ctrl and RKV-supplemented mice, assessed by a bioluminescence assay. Representative experiment out of 2 independent experiments. **(E)** Survival of Ctrl and RKV-supplemented mice after infection. Pool of 2 independent experiments. (A and C) Two-tailed Mann-Whitney, (D) Two-way ANOVA with post-test Bonferroni, (E) Log-Rank Mantel-Cox test. Error bars represent SEM. ** $p < 0.01$ and *** $p < 0.001$.

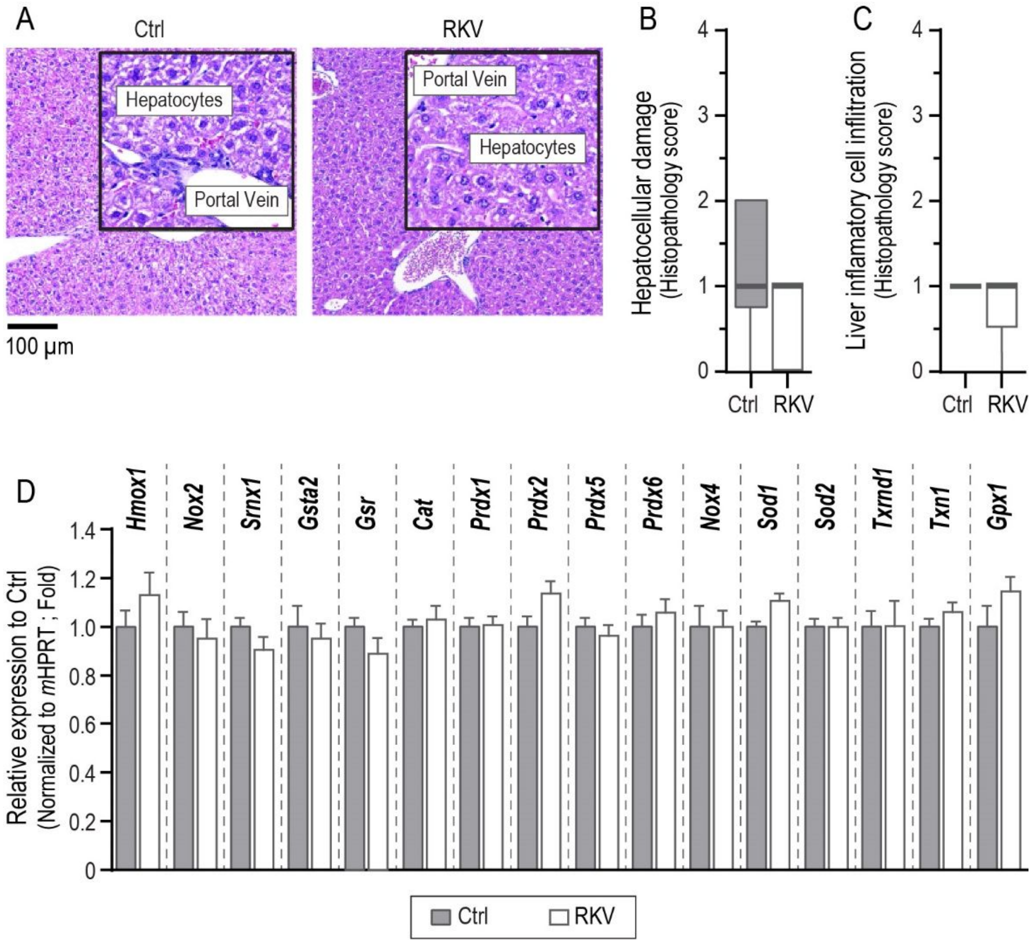

Figure S2 (Related to Table 1)– RKV supplementation does not cause liver toxicity or an increased inflammatory state. (A) Representative microphotographs of liver sections from Ctrl and RKV-supplemented mice stained with HE. Insets show a normal aspect and organization of the liver cells. **(B)** Hepatocellular damage score of livers from Ctrl and RKV-supplemented mice. **(C)** Hepatic inflammatory cell infiltration score in livers from Ctrl and RKV-supplemented mice at 39 hpi. **(B** and **C)** One experiment with 5 mice per group. **(D)** Expression of several oxidative stress-related genes in whole livers of Ctrl and RKV-supplemented mice at 46 hpi. Pool of 3 independent experiments. Two-tailed Mann-Whitney test. Error bars represent SEM.

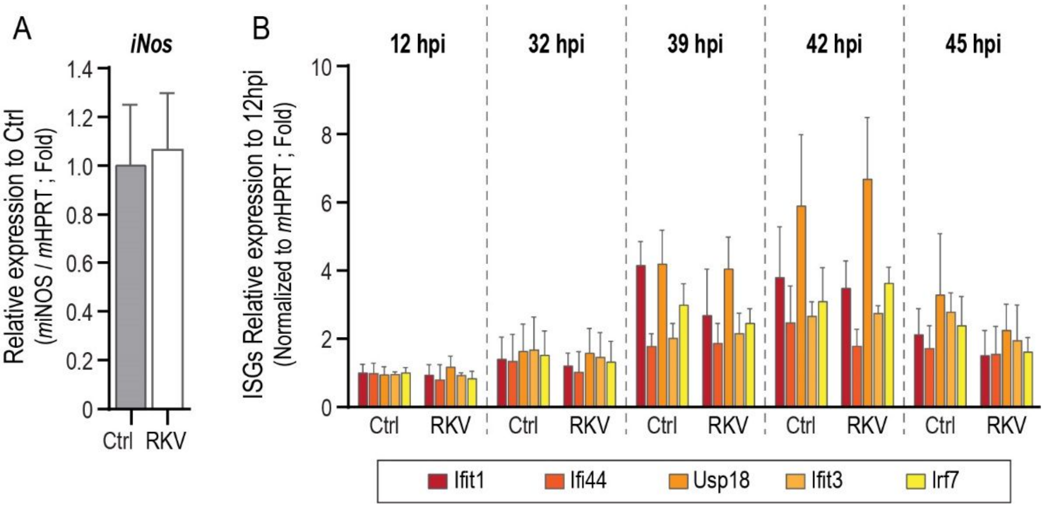

Figure S3 (Related to Figure 4)– The effect of RKV supplementation on liver parasite load is not dependent on increased expression of iNOS or of interferon-stimulated genes (ISGs) (A) Expression of iNOS in whole livers of Ctrl and RKV-supplemented mice at 46 hpi. Pool of 3 independent experiments. Unpaired t-test. **(B)** Expression of five ISGs in RKV-supplemented and Ctrl mice at different timepoints during *P. berghei* liver infection. Pool of 2 – 4 independent experiments. Kruskal-Wallis with post-test Dunn's. Error bars represent SEM.

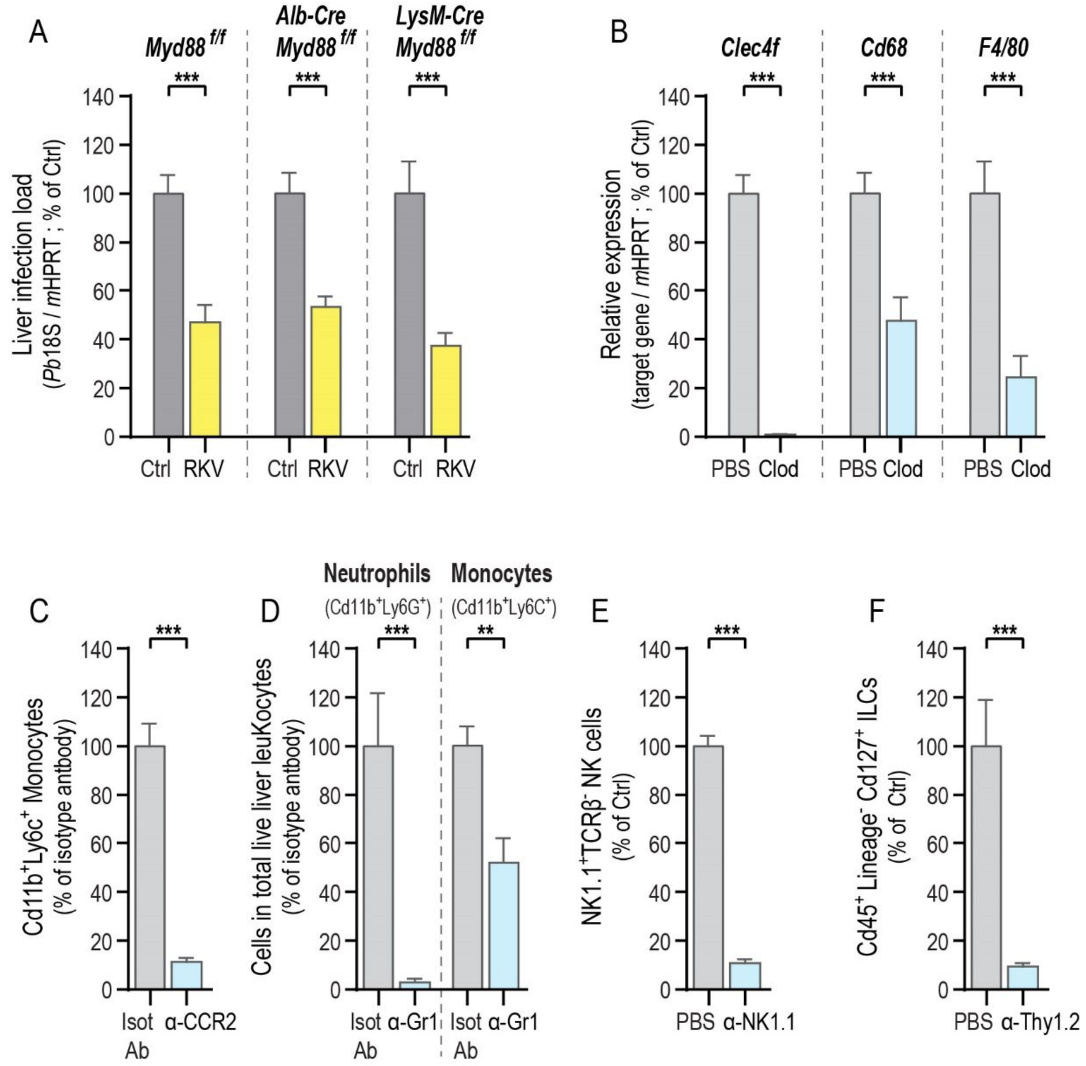

Figure S4 (Related to Figure 5)– RKV-dependent parasite elimination is dependent on the immune system but is not singly mediated by either hepatocytes, macrophages, monocytes, neutrophils, NK cells or ILCs. (A) Alb-Cre.MyD88$^{f/f}$, which lack MyD88 specifically on hepatocytes, LysM-Cre.MyD88$^{f/f}$, which lack MyD88 specifically on myeloid cells, and the corresponding littermate control mice (MyD88$^{f/f}$) were allowed to drink Ctrl and RKV supplemented water before infection with *Plasmodium* sporozoites. Liver load was assessed at 46 hpi. Pool of > 3 and 2 independent experiments, for Alb-Cre.MyD88$^{f/f}$ and LysM-Cre.MyD88$^{f/f}$, respectively. **(B)** The efficiency of the depletion of phagocytes by clodronate administration was assessed at 46 hpi by qRT-PCR, by quantifying the expression of Clec4f (a marker of Kupffer cells), CD68 (a marker of monocytes and macrophages) and F4/80 (a marker of macrophages) in whole livers. Pool of 2 independent experiments. **(C)** The efficiency of the depletion of monocytes by anti-CCR2 administration was assessed at 46 hpi by flow cytometry. After extraction, liver leukocytes were stained with LIVE/DEAD

Fixable Aqua Dead Cell Staining kit, anti-CD11b, anti-CD11c, anti-Ly6C and anti-Ly6G. Monocytes were defined as $Ly6C^{+}$ $Ly6G^{-}$ cells inside the $CD11b^{+}$ $CD11c^{-}$ population. One experiment. **(D)** The efficiency of the depletion of neutrophils and monocytes by anti-Gr1 administration was assessed at 46 hpi by flow cytometry. Total liver leukocytes were stained with LIVE/DEAD Fixable Aqua Dead Cell Staining kit, anti-CD11b, anti-CD11c, anti-Ly6C and anti-Ly6G. Neutrophils were defined $Ly6G^{+}$ $Ly6C^{-}$ cells and monocytes as $Ly6C^{+}$ $Ly6G^{-}$ cells, both inside the $CD11b^{+}$ $CD11c^{-}$ population. Representative experiment out of 3 independent experiments. **(E)** The efficiency of the depletion of NK cells by anti-NK1.1 administration was assessed at 46 hpi by flow cytometry. Liver leukocytes were stained with LIVE/DEAD Fixable Aqua Dead Cell Staining kit, anti-NK1.1 and anti-TCRβ. NK cells were defined as $NK1.1^{+}$ $TCR\beta^{-}$ cells inside the total live population. Representative experiment out of 3 independent experiments. **(F)** The efficiency of the depletion of ILCs by administration of anti-Thy1.2 to $Rag2^{-/-}$ mice was assessed at 46 hpi by flow cytometry. Total liver leukocytes were stained with LIVE/DEAD Fixable Aqua Dead Cell Staining kit, anti-CD45, anti-CD3ε, anti-Gr1, anti-CD11b, anti-CD11c, anti-B220, anti-Ter119 and anti-CD127. ILCs were defined as $Lineage^{-}$ $CD127^{+}$ cells inside the live $CD45^{+}$ population. Lineage: anti-CD3ε, anti-Gr1, anti-CD11b, anti-CD11c, anti-B220 and anti-Ter119. One experiment. Error bars represent SEM. **(A, C** and **F)** Two-tailed Mann-Whitney test; **(B**, **D** and **E)** Unpaired t-test. ** $p < 0.01$ and *** $p < 0.001$.

Table S1 (Related to Figures 1-5)- List of primer sequences used for gene expression quantification.

Gene	forward primer (5' - 3')	reverse primer (5' - 3')
Pb18S	AAGCATTAAATAAAGCGAATACATCCTTAC	GGAGATTGGTTTTGACGTTTATGTG
Hprt	TTTGCTGACCTGCTGGATTAC	CAAGACATTCTTTCCAGTTAAAGTTG
Ifit1	CCTTTACAGCAACCATGGGAGA	GCAGCTTCCATGTGAAGTGAC
Ifi44	TCGATTCCATGAAACCAATCAC	CAAATGCAGAATGCCATGTTTT
Usp18	CGTGCTTGAGAGGGTCATTTG	GGTCGGGAGTCCACAACTTC
Ifit3	CTGAACTGCTCAGCCCACAC	TGGACATACTTCCTTCCCTGA
Irf7	CTTCAGCACTTTCTTCCGAGA	TGTAGTGTGGTGACCCTTGC
Hamp	CCTATCTCCATCAACAGATG	AACAGATACCACACTGGGAA
Hmox1	GTCTCTGCAGGGGCAGTATC	TGCTCGAATGAACACTCTGG
Prdx1	GTTGGCCGCTCTGTGGATGAGAT	ATCACTGCCAGGTTTCCAGCCAGC
Prdx2	GTTCTCCGGCCTAGGGCTCTCTC	GCCGGAGGCCATGACTGCGTG
Prdx5	GCTCCGTGCATCGACGTGCT	CTCCCACCTTGATCGGGGCCA
Prdx6	CACCACGGGCAGGAACTTTGATG	TCACGCTCTCTCCCTTCTTCCAGT
Nox2	TGCAGTGCTATCATCCAAGC	CTTTCTCAGGGGTTCCAGTG
Nox4	TCAGGACAGATGCAGATGCT	CTGGAAAACCTTCCTGCTGT
Sod1	TACTGATGGACGTGGAACCC	GAACCATCCACTTCGAGCA
Sod2	GCTTGATAGCCTCCAGCAAC	ACTGAAGTTCAATGGTGGGG
Cat	TCAGGGCCGCCTTTTTGCCT	ACTCGAGCGCGGTAGGGACA
Txrnd1	ATGGACAGTCCCATCCCGGGA	GCCCACGACACGTTCATCGTCT
Txn1	TGCTACGTGGTGTGGACCTTGC	TCTGCAGCAACATCCTGGCAGT
Srnx1	AGTAGTAGTCGCCACCCTGG	AGAGCCTGGTGGACACGAT
Gsta2	TTGAAGTAGTGAAGCACGGG	ATTGGGAGCTGAGTGGAGAA
Gpx1	CAATGTAAAATTGGGCTCGAA	GTTTCCCGTGCAATCAGTTC
Gsr	ATCGTGCATGAATTCCGAGT	GGTGGTGGAGAGTCACAAGC
Clec4f	TGAGTGGAATAAAGAGCCTCCC	TCATAGTCCCTAAGCCTCTGGA
Cd68	AGCTGCCTGACAAGGGACACT	AGGAGGACCAGGCCAATGAT
F4/80	CCCCAGTGTCCTTACAGAGTG	GTGCCCAGAGTGGATGTCT

TRANSPARENT METHODS

Chemicals

RPMI 1640, RPMI 1640 without arginine, William's E, PBS pH 7.4, trypsin, FBS, non-essential amino acids, penicillin/streptomycin, glutamine, HEPES pH 7, liver perfusion medium (LPM) and liver digestion medium (LDM) were purchased from Gibco/Invitrogen. L-arginine hydrochloride, L-lysine and L-valine were purchased from FisherScientific. All other chemicals were obtained from Sigma, unless otherwise specified.

Cells

Huh7 cells were cultured in RPMI 1640 medium supplemented with 10% v/v FBS, 1% v/v non-essential amino acids, 1% v/v penicillin/streptomycin, 1% v/v Glutamine and 1% v/v HEPES, pH 7 and maintained at 37 °C with 5% CO_2. Mouse primary hepatocytes were cultured in William's E medium supplemented with 4% v/v FBS and 1% v/v penicillin/streptomycin and maintained at 37 °C with 5% CO_2.

Mice

All animals used in this study were housed in the facilities of Instituto de Medicina Molecular João Lobo Antunes (iMM, Lisbon, Portugal), with a maximum of five animals per cage, and free access to water and food. C57BL/6J wild-type (WT) mice were purchased from Charles River Laboratories (L'Arbresle, France). Nos2$^{-/-}$ mice were purchased from The Jackson Laboratory (Bar Harbor, ME, USA). Ifnar$^{-/-}$, MyD88$^{-/-}$ and Rag2$^{-/-}$ experimental mice, and Alb-Cre and LysM-Cre breeders were purchased from Instituto Gulbenkian de Ciência (IGC, Lisbon, Portugal). All other mouse strains were obtained from breedings established at iMM's rodent facility. Four weeks old male mice were used in all experiments that employed exclusively C57BL/6J WT mice. Both male and female 4 to 8 weeks old mice were used in experiments that employed genetically deficient mice. In the latter experiments, 4 to 8 weeks old WT or littermate male and/or female mice were used to match gender and age of the genetically deficient mice. All animal experiments were performed in strict compliance to the

guidelines of iMM´s animal ethics committee (ORBEA) and the Federation of European Laboratory Animal Science Associations (FELASA). In the experiments in which the infection was allowed to proceed to the blood, animals were humanely euthanized at the first behavioral signs of onset of experimental cerebral malaria (ECM).

Parasites

Sporozoites were isolated from the salivary glands of infected female *Anopheles stephensi* mosquitoes bred at iMM's insectary facility, prior to being employed in infections. A GFP/luciferase-expressing *P. berghei* ANKA parasite line (676m1cl1 line) was used in all experiments (Ploemen et al., 2009), with the exception of the flow cytometry experiments, in which a GFP-expressing *P. berghei* ANKA parasite line (259cl1 line) was employed (Franke-Fayard et al., 2004) and the *P. yoelii* experiment, in which a GFP-expressing *P. yoelii* (strain 17XNL) was employed (Ono et al., 2007).

Isolation of mouse primary hepatocytes

Mouse primary hepatocytes were isolated using a modified two-step perfusion protocol followed by a Percoll purification step, as previously described (Goncalves et al., 2007, Liehl et al., 2014, Meireles et al., 2017). Mice were euthanized by CO_2 inhalation and immediately processed for cannulation of the portal vein using a 26-gauge needle, followed by the sectioning of the inferior vena cava (IVC) to allow the fluid to drain. The liver was perfused with liver perfusion medium (LPM), followed by liver digestion medium (LDM). Intermittent clamping of the IVC was performed during LDM perfusion to improve tissue digestion. After digestion, the liver was excised and the cells were liberated, sequentially filtered through a 100 µm and a 70 µm cell strainer and spun at 50×g for 3 min. The pellet was resuspended in William's Medium E with 10% v/v of FBS, carefully overlaid on a 60% v/v Percoll solution (1:1) and spun at 750×g for 20 min, without break, at 20 °C. Viable hepatocytes deposited in the pellet were washed with William's E Medium with 10% v/v FBS, spun at 50×g for 3 min and resuspended in complete William's E Medium (supplemented with 4% v/v FBS and 1% v/v

penicillin/streptomycin). Hepatocytes were then plated at a density of 1.0 × 10^5 in 24-well plates. Viability and yield were assessed by trypan blue staining.

In vitro and *ex vivo* amino acid supplementation

In the conditions with physiological concentrations of Arg, the medium of Huh7 cells or mouse primary hepatocytes was replaced by Arg-free medium supplemented with 100 µM Arg. In the conditions in which there is supplementation of Arg, the approximate supraphysiological concentration of this amino acid which is normally present in RPMI, 1 mM, was added. Supplementations with Lys and Val, were performed by adding 20 mM of each of these amino acids to medium with physiological (K, V and KV) or supraphysiological concentrations of Arg (RK, RV, RKV). These concentrations of Lys and Val were chosen because they have been shown to completely inhibit arginase activity in rat primary hepatocytes at physiological concentrations of Arg (Lerzynski et al., 2006).

Overall *in vitro* infection by luminescence

Overall hepatic infection was determined by measuring the luminescence intensity in Huh7 cells infected with the above referred GFP/luciferase-expressing *P. berghei* line, as previously described (Ploemen et al., 2009). Huh7 cells (1.0 × 10^4 per well) were seeded in 96-well plates the day before infection. Sporozoite addition was followed by centrifugation at 1800xg for 5 min. Medium was replaced approximately 2 hpi by the appropriate medium. Parasite infection load was measured 48 hpi by a bioluminescence assay (Biotium) using a multiplate reader Infinite M200 (Tecan). Cell viability was assessed by the CellTiter-Blue assay (Promega) according to the manufacturer’s protocol.

Quantification of *P. berghei* parasite numbers and development by flow cytometry

Intracellular parasite numbers and development were assessed by determining the percentage of GFP^+ cells and by measuring the intensity of the GFP signal of mouse

primary hepatocytes infected with a GFP-expressing *P. berghei* line at 48 hpi, as previously described (Prudencio et al., 2008). Primary hepatocytes (1.0×10^5 per well) were infected with 5.0×10^4 sporozoites one day after being plated and the medium was replaced by the appropriate medium 2 h after infection. Cells were collected for flow cytometry analysis at 48 hpi and analyzed on a Becton Dickinson FACSCalibur. Data acquisition and analysis were carried out using the CELLQuest (version 3.1.1 f1, Becton Dickinson) and FlowJo (version 6.4.7, FlowJo) software packages, respectively.

In vivo amino acid supplementation

The drinking water of 4 to 8 weeks old mice, was replaced by sterilized water containing 2.5% (w/v) L-arginine hydrochloride (FisherScientific), 2.5% (w/v) L-lysine (FisherScientific), 2.5% (w/v) L-valine (FisherScientific) or a combination of the three amino acids (RKV). The mice were allowed to drink *ad libitum* for 4 weeks, unless otherwise specified. Non-supplemented, sterilized water was provided to Ctrl mice. The supplementation was maintained until completion of the experiment.

In vivo treatments

C57BL/6J WT mice were lethally irradiated (900 rad) in an Irradiator Gammacell ELAN 3000, one day before *P. berghei* sporozoite injection. For depletion of phagocytic cells, 200 µL of liposome-encapsulated clodronate (Clodronate Liposomes) were injected i.v. 2 days before infection. Mice injected with 200 µL of liposome-encapsulated PBS were used as controls. Monocytes were depleted by the i.p. injection of 20 µg of anti-CCR2 antibody (clone MC-21; kindly provided by Matthias Mack (Mack et al., 2001)) daily from day -2 to day 1 post-infection. Two hundred and fifty µg of anti-Gr1 (clone RB6-8C5; BioXCell) were injected i.p. 2 h after infection, to deplete both neutrophils and monocytes. For NK cell depletion, 150 µg of anti-NK1.1 antibody (clone PK136; BioXCell) were injected i.p. 1 day prior to infection. Finally, to deplete ILCs, $Rag2^{-/-}$ mice were injected i.p. with 200 µg of anti-Thy1.2 antibody (clone 30H12;

kindly provided by Marc Veldhoen, iMM), 1 day before infection. Ctrl mice were injected with PBS, or with IgG2a or IgG2b isotype control antibodies (clones 2A3 and LTF-2, respectively; BioXCell).

In vivo infection and liver collection

Mice were infected intravenously, through retro-orbital injection of 1.0×10^4 *P. berghei* sporozoites. Livers were collected at 46 hours post-infection (hpi), unless otherwise specified, and homogenized in 3 mL of denaturing solution (4 M guanidine thiocyanate; 25 mM sodium citrate pH 7, 0.5% w/v *N*-lauroylsarcosine and 0.7% v/v β mercaptoethanol in DEPC-treated water). In some experiments, one of the liver lobes was fixed in 4% paraformaldehyde (PFA) for immunofluorescence or histopathology analyses. In experiments in which immune cell populations were depleted, a section of the liver was used to extract total liver leukocytes, as described below.

Bioluminescence analyses of *in vivo* hepatic infection and blood parasite load

In vivo hepatic parasite load was determined by real-time *in vivo* imaging of the infected mice at 45-47 hpi, using the *in vivo* IVIS Lumina Imaging System, as previously described (Ploemen et al., 2009). Briefly, animals were anesthetized using isofluorane to allow the subcutaneous (s.c.) injection (in the neck) of 200 µL D-luciferin (PerkinElmer) dissolved in PBS (10 mg/ml). After 5 min, animals were anesthetized through intraperitoneal (i.p.) injection of a solution of Ketamine/Xylazine to allow the bioluminescent measurements, which were performed around 10 min after the injection of D-luciferin. Before every experiment, the belly of the animals was shaved to allow for a better detection of the bioluminescent signal.

The presence of luciferase-expressing erythrocytic-stage parasites was monitored daily, from day 2 after infection, as described in (Zuzarte-Luis et al., 2014). Five µL of blood was collected from the tail vein into 45 µL of lysis buffer (Firefly Luciferase Kit, Biotium). Luminescence was determined by adding 50 µL of D-luciferin dissolved in firefly luciferase assay buffer (FLAB) to 30 µL of lysate, according to the manufacturer's instructions, and immediately measured using

a multiplate reader (Tecan, Switzerland). Values of luciferase activity were expressed as relative luminescence units (RLU).

RNA extraction, cDNA synthesis and qRT-PCR analysis of hepatic infection

Total RNA was extracted from liver homogenates using the TripleXtractor Direct RNA kit (Grisp), according to the manufacturers´ instructions. Complementary DNA (cDNA) was synthesized from 1 µg of RNA using the NZY First-Strand cDNA synthesis kit (NZYtech), according to the manufacturer´s instructions. The qRT-PCR reaction was performed in a total volume of 10 µL in a ViiA 7 Real-Time PCR system (Applied Biosystems) using the iTaq^{TM} Universal SYBR® Green kit (BioRad). Parasite load was quantified using primers specific to *P. berghei* 18S RNA (Table S1). The expression of the mouse housekeeping gene hypoxanthine-guanine phosphoribosyltransferase (*Hprt)* was used for normalization in all experiments (Table S1). Analysis of qRT-PCR data was performed using the delta-delta CT relative quantification method.

Immunohistochemical staining of liver sections

For microscopy, PFA-fixed liver lobes were cut in 50 µm sections and were incubated in permeabilization/blocking solution (1% w/v BSA, 0.5% v/v Triton-X100 in PBS) at room temperature for 1 h, followed by a 2 h incubation at room temperature with an anti-UIS4 antibody (dilution 1:500). Liver sections were further incubated in a 1:300 dilution of anti-GFP-Alexa488 antibody (Invitrogen) and anti-goat Alexa-Fluor 568 (Invitrogen) in the presence of a 1:1000 dilution of Hoechst 33342 (Invitrogen) and a 1:100 dilution of Phalloidin-660 (Invitrogen) for actin staining for 1 h. After washing, the liver sections were mounted on microscope slides with Fluoromount (SouthernBiotech). Widefield images for size determination were acquired in a Zeiss Axiovert 200M microscope. Images were processed with ImageJ software (version 1.47).

Histology analyses

Mice were sacrificed by CO_2 narcosis. Necropsy was performed and liver and kidneys were collected, fixed in 10% neutral buffered formalin and processed for routine histology. Four µm sections were stained with hematoxylin and eosin (HE), and analyzed by a pathologist blinded to experimental groups on a Leica DM2000 microscope coupled to a Leica MC170 camera. Lesions were recorded (diagnosed according to previously published criteria (Thoolen et al., 2010, Frazier et al., 2012)), and inflammation was scored according to a 5-tier severity scale (0, absent; 1, minimal; 2, mild; 3, moderate; 4, marked).

Plasma biochemistry

Blood was collected terminally by cardiac puncture and plasma was separated from the cells by centrifugation (4000xg, 10 min, 4˚C). The following parameters were measured in Ctrl and RKV-supplemented mouse plasma (DNAtech, Lisbon, Portugal): Alanine aminotransferase (ALT), Alkaline phosphatase (ALP), Aspartate aminotransferase (AST), Total protein, Total bilirrubin, Blood urea nitrogen (BUN), Gamma-glutamyltransferase (GGT), and Creatinine.

Isolation of liver leukocytes

Livers were dissociated in a PBS solution containing DNase (2 U per mL), filtered through a 100 µm cell strainer, and centrifuged at 400xg for 5 min. Liver leukocytes were purified by centrifugation using 10 mL of a 35% (v/v) Percoll (Sigma) solution, followed by a centrifugation at 20 °C, 1360xg for 20 min, without break or acceleration. After one wash, RBCs were lysed with 3 mL Ammonium-Chloride-Potassium (ACK) buffer (155 mM Ammonium chloride; 10 mM Potassium bicarbonate; and 0.1 mM Ethylenediaminetetraacetic acid (EDTA) in DEPC-treated water) for 3 min at room temperature (RT). Lysis was stopped by adding PBS with 2% (v/v) foetal bovine serum (FBS, Gibco) and after a final centrifugation at 400xg for 5 min, the cells were resuspended in 200 µL of 2% FBS in PBS for extracellular FACS staining.

Extracellular FACS staining

For surface staining, cells were Fc blocked with anti-CD16/CD32 (clone 93; eBioscience) and incubated for 20 minutes on ice with antibodies and LIVE/DEAD Fixable Aqua Dead Cell Staining kit (Molecular Probes) in PBS. The following monoclonal antibodies were used: anti-CD3ε (clone 145-2C11; BD Biosciences), anti-CD11b (clone M1/70; Biolegend), anti-CD11c (clone N418; Biolegend), anti-CD45 (clone 30-F11; Biolegend), anti-Ly6C (clone HK1.4; Biolegend), anti-Ly6G (clone 1A8; Biolegend), anti-NK1.1 (clone PK136; Biolegend), anti-TCRβ (clone H57-597; Biolegend), anti-Gr1 (clone RB6-8C5; eBioscience), anti-B220 (clone RA3-6B2; Biolegend), anti-Ter119 (clone TER-119; Biolegend), and anti-CD127 (clone A7R34; Biolegend). Cells were acquired on a LSR Fortessa X-20 cytometer (BD Biosciences) with the FACSDiva software (version 6.2, BD), and data was analyzed using the FlowJo software.

Statistical Analyses

Statistical analyses were performed using the GraphPad Prism 5 software. All datasets were analyzed for normality with the D'Agostino and Pearson omnibus or the Kolmogorov-Smirnov normality tests prior to statistical analyses. Kruskal-Wallis, One-way ANOVA, Two-way ANOVA, Two-tailed Mann-Whitney test, Unpaired t-test, or Log-Rank Mantel-Cox test were used for significance of the differences observed, as indicated in each figure. ns – not significant, * $p < 0.05$, ** $p < 0.01$ and *** $p < 0.001$.
